# Zinc(II) binding on human wild-type ISCU and Met140 variants modulates NFS1 desulfurase activity

**DOI:** 10.1016/j.biochi.2018.07.012

**Published:** 2018-09

**Authors:** Nicholas G. Fox, Alain Martelli, Joseph F. Nabhan, Jay Janz, Oktawia Borkowska, Christine Bulawa, Wyatt W. Yue

**Affiliations:** a Structural Genomics Consortium, Nuffield Department of Clinical Medicine, University of Oxford, OX3 7DQ, UK; b Pfizer Rare Disease Research Unit, Worldwide Research and Development, Pfizer Inc., 610 Main Street, Cambridge, MA, 02139, United States

**Keywords:** Cysteine desulfurase, Friedreich's ataxia, Iron-sulfur cluster, ISCU, Zinc, ACP, acyl carrier transfer protein, BLI, BioLayer interferometry, BSA, bovine serum albumin, CD, circular dichroism, DMPD, N,N-dimethyl-*p*-phenylenediamine, DSF, differential scanning fluorimetry, DTT, dithiothreitol, EDTA, ethylenediaminetetracetic acid, Fe-S, iron sulfur, FRDA, Friedreich's ataxia, FXN, frataxin, HEPES, 4-(2-hydroxyethyl)-1-piperazineethanesulfonic acid, IPTG, isopropyl β-D-1-thiogalactopyranoside, PLP, pyridoxal 5′-phosphate, SDA, protein complex composed of NFS1, ISD11 and ACP, SDAU, protein complex composed of NFS1, ISD11, ACP, and ISCU, SDAUF, protein complex composed of NFS1, ISD11, ACP, ISCU, and frataxin, TCA, trichloroacetic acid, TCEP, tris(2-carboxyethyl) phosphine, Tris, tris(hydroxymethyl)aminomethane

## Abstract

Human *de novo* iron-sulfur (Fe-S) assembly complex consists of cysteine desulfurase NFS1, accessory protein ISD11, acyl carrier protein ACP, scaffold protein ISCU, and allosteric activator frataxin (FXN). FXN binds the NFS1-ISD11-ACP-ISCU complex (SDAU), to activate the desulfurase activity and Fe-S cluster biosynthesis. In the absence of FXN, the NFS1-ISD11-ACP (SDA) complex was reportedly inhibited by binding of recombinant ISCU. Recent studies also reported a substitution at position Met141 on the yeast ISCU orthologue Isu, to Ile, Leu, Val, or Cys, could bypass the requirement of FXN for Fe-S cluster biosynthesis and cell viability. Here, we show that recombinant human ISCU binds zinc(II) ion, as previously demonstrated with the *E. coli* orthologue IscU. Surprisingly, the relative proportion between zinc-bound and zinc-depleted forms varies among purification batches. Importantly the presence of zinc in ISCU impacts SDAU desulfurase activity. Indeed, removal of zinc(II) ion from ISCU causes a moderate but significant increase in activity compared to SDA alone, and FXN can activate both zinc-depleted and zinc-bound forms of ISCU complexed to SDA. Taking into consideration the inhibition of desulfurase activity by zinc-bound ISCU, we characterized wild type ISCU and the M140I, M140L, and M140V variants under both zinc-bound and zinc-depleted conditions, and did not observe significant differences in the biochemical and biophysical properties between wild-type and variants. Importantly, in the absence of FXN, ISCU variants behaved like wild-type and did not stimulate the desulfurase activity of the SDA complex. This study therefore identifies an important regulatory role for zinc-bound ISCU in modulation of the human Fe-S assembly system *in vitro* and reports no ‘FXN bypass’ effect on mutations at position Met140 in human ISCU. Furthermore, this study also calls for caution in interpreting studies involving recombinant ISCU by taking into consideration the influence of the bound zinc(II) ion on SDAU complex activity.

## Highlights

• Recombinantly expressed human ISCU varies in amount of bound zinc between batches.
• Zinc stabilizes ISCU, but inhibits NFS1 desulfurase activity.
• A frataxin bypass in the human Fe-S complex for ISCU variants was not observed.

## 1 Introduction

Iron-sulfur (Fe-S) clusters are prosthetic groups required for critical cellular functions including oxidative respiration, DNA repair, and biosynthesis of other cofactors [1,2]. The Fe-S biosynthetic pathway in humans is located in the mitochondrial matrix and is initiated by a protein complex of NFS1, ISD11, ISCU and frataxin (FXN) [[3], [4], [5], [6]]. Recently, this protein complex was shown to harbor an additional component, the acyl carrier protein (ACP, also known in human as NDUFAB1) in recombinant preparations [7,8], whether ACP is an intrinsic component of the complex in vivo remains to be determined but has been suggested to coordinate mitochondrial fatty acid synthesis to iron sulfur cluster biogenesis [9]. Within this complex (SDAUF), the cysteine desulfurase NFS1 (homolog of yeast Nfs1 or bacterial IscS) catalyzes the pyridoxal phosphate (PLP)-dependent conversion of l-cysteine to l-alanine, and generates a persulfide species that delivers the sulfane sulfur to the Fe-S scaffold protein, ISCU (homolog of yeast Isu or bacterial IscU) [10,11]. The eukaryotic-specific ISD11 (LYRM4), belonging to the Leu-Tyr-Arg (LYR) superfamily of small basic proteins complexed to ACP [9], interacts with and stabilizes NFS1, potentially regulating the desulfurase activity [7,[12], [13], [14], [15], [16], [17]]. Frataxin (FXN, homolog of yeast Yfh1 or *E. coli* CyaY) acts as an allosteric regulator of the Fe-S assembly complex. Human FXN is shown to stimulate the rate of cysteine desulfurase activity [18] and iron sulfur cluster biosynthesis [19] *in vitro*, whereas bacterial CyaY appears to inhibit the counterpart desulfurase IscS [20].

During *de novo* Fe-S assembly, ISCU serves as the scaffold, obtaining ferrous iron and inorganic sulfide to assemble the Fe-S cluster. Human ISCU has three conserved cysteine residues, namely Cys69, Cys95, and Cys138 (also referred as Cys35, Cys61, and Cys104 respectively in the literature^1^), involved in coordinating the Fe-S cluster. The LPPVK motif on ISCU is then recognized by the chaperone protein GRP75, to deliver the Fe-S cluster either to an apo-protein target, or possibly into the 4Fe-4S cluster pathway [21]. A wealth of bacterial IscU structures determined from NMR and X-ray crystallography [[22], [23], [24], [25], [26], [27], [28], [29], [30], [31]] have revealed two conformational states of the protein with respect to its secondary structures and local environment surrounding the conserved cysteines. The two conformations, namely structured and disordered, were observed with different liganded or mutant ISCU proteins, suggesting they may play a role in ISCU function and interaction.

Expansion of a GAA trinucleotide repeat in the FXN gene results in cellular depletion of frataxin protein and causes the autosomal recessive neurodegenerative disease Friedreich's ataxia (FRDA). Recently, a “suppressor” mutation in *S. cerevisiae* Isu, corresponding to the substitution of Met141 (also referred as Met107 in the literature^∗^) to either an Ile, Leu, Val, or Cys, was shown to increase cell viability and rescue Fe-S cluster synthesis in Yfh1 (FXN equivalent)-deleted cells [[32], [33], [34]]. The IscU suppressor variant was also shown to bind and activate Nfs1 *in vitro* using purified yeast proteins, hence bypassing the requirement for Yfh1 [35]. Phylogenetic analysis of ISCU sequences showed that in prokaryotes, the equivalent position to yeast Met141 is more commonly an Ile, Leu, Val or Cys. Therefore, at the amino acid sequence level, substitution of Met141 with the aforementioned residues likely yielded the yeast Isu suppressor variant that behaved like the prokaryotic orthologue in facilitating cluster assembly without frataxin [33]. Also, transfer of the persulfide from NFS1 to the proposed sulfur acceptor (i.e. ISCU Cys138) [36,37] is positioned only two residues away from ISCU Met140 (human numbering) and a bacterial structure of IscU has shown to adopt a conformation change around this region when a [2Fe-2S] cluster is bound [27]. A substitution of the nearby Met residue could therefore impose a direct effect on this crucial functional interaction.

The objectives of this study are two-fold, first to investigate the biochemical and biophysical properties of recombinant human ISCU towards its functional interaction within the SDAUF complex for Fe-S cluster assembly, and second to determine if the recombinant Met140 substitution variants of human ISCU result in frataxin bypass *in vitro*. Along this study, we also uncovered a role for zinc(II) ion, co-purified with recombinant ISCU wild-type and variant proteins, on NFS1 desulfurase activity.

## 2 Experimental procedures

### 2.1 Cloning, expression and purification of human ISCU, FXN, and NFS1-ISD11

Constructs of human ISCU (Δ1-34) and Frataxin (Δ1-80) were subcloned into the pNIC28-Bsa4 vector (GenBank ID: EF198106) for recombinant *E. coli* expression using BL21 (DE3)-R3-pRARE2 cells. For bi-cistronic co-expression of NFS1-ISD11, a DNA fragment encoding His-tagged ISD11 and non-tagged NFS1 (Δ1-55), separated by an in-frame ribosomal binding site, was sub-cloned into pNIC28-Bsa4 vector. ISCU variants (M140I, M140L, and M140V) were constructed using the QuikChange site-directed mutagenesis kit (Strategene), and confirmed by sequencing of plasmid DNA and intact mass spectrometry of purified proteins. Cells transformed with the above plasmids were grown in Terrific Broth and induced with 0.1 mM isopropyl β-D-1-thiogalactopyranoside (IPTG) for 16 h at 18 °C. Cell pellets were resuspended in binding buffer (50 mM HEPES pH 7.5, 500 mM NaCl, 20 mM Imidazole, 5% glycerol, and 2 mM TCEP) containing EDTA-free protease inhibitor (Merck), and lysed by sonication. For NFS1-ISD11 purification, 150 μM pyridoxal 5′-phosphate (PLP) was supplemented to the binding buffer during sonication. The clarified supernatant was incubated with 2.5 mL Ni Sepharose 6 fast flow resin (GE Healthcare), washed and eluted with binding buffer containing 40 mM and 250 mM Imidazole, respectively. Elution fractions were collected, 10 mM DTT was added to samples containing ISCU or NFS1, and loaded onto gel filtration (Superdex S75 for ISCU and FXN, Superdex S200 for NFS1-ISD11 complex; GE Healthcare). Peak fractions were collected, treated with His-TEV protease to remove His-tag, and then passed onto Ni Sepharose 6 fast flow resin to remove His-TEV and cleaved His-tag. Fractions containing target protein were collected and buffer exchanged into gel filtration buffer (50 mM HEPES pH 7.5, 200 mM NaCl, 5% glycerol, and 2 mM TCEP). As reported by others, recombinantly expressed NFS1-ISD11 complex co-purified with *E. coli* ACP, and will hereafter be referred to as the NFS1-ISD11-ACP (SDA) complex.

### 2.2 Methylene blue activity assay

Sulfide production, due to cysteine desulfurase enzyme activity, was measured using the methylene blue colorimetric assay as described previously [3,38]. The standard assay was performed in buffer consisting of 50 mM HEPES pH 7.5, 200 mM NaCl, 10 mM DTT and either 100 μM EDTA or 50 μM ZnCl_2_. When noted, concentration of NFS1-ISD11-ACP (SDA) was at 0.5 μM, ISCU (U) at 2.5 μM, and Frataxin (F) at 40 μM was mixed in a 1.5 mL black Eppendorf tube with total volume of 800 μL. To allow comparison, the same excess of ISCU (5 equivalents to [SDA]) and FXN (80 equivalents to [SDA]) were used for all wild-type and mutant ISCU. The reaction was initiated by adding 100 μM L-Cysteine and placed in 37 °C incubator for 10 min (with FXN) or 20 min (without FXN) and then quenched with 100 μL of 30 mM FeCl_3_ in 1.2 N HCl and 100 μL of 20 mM N*,N*-dimethyl-*p*-phenylenediamine (DMPD) in 7.2 N HCl and placed back in 37 °C incubator for 20 min, followed by centrifugation to spin down precipitant and then take the absorbance at 670 nm. Concentration of sulfide was calculated *via* a standard curve of Na_2_S. Concentrations for ISCU and FXN were determined by a titration until maximum flux in activity was seen. To determine IC_50_ of zinc(II) ion inhibition of the SDAU complex, ISCU was treated with EDTA (10 fold excess to [ISCU]) to remove any zinc and then buffer exchanged into EDTA-free buffer to remove the EDTA. The methylene blue assay was then used in the presence of buffer containing a serial dilution of zinc concentrations and plotting [inhibitor] *vs.* response.

### 2.3 Complex reconstitution by size exclusion chromatography

Reconstitution of the recombinant NFS1-ISD11-ISCU complex was mediated by co-expression of all three proteins in a poly-cistronic fashion, where only ISD11 is His-tagged. Expression and affinity chromatography were carried out as described above for single proteins. Complex-containing fractions eluted from affinity step were pooled, and analytical gel filtration was performed using a Dionex Ultimate ™ 3000 system. The Sepax SRT SEC-300 7.8 × 300 mm column was pre-equilibrated in buffer containing 50 mM HEPES pH 7.5, 150 mM NaCl, 5% glycerol and 2 mM TCEP, and run at 0.5 mL/min. Complex formation was confirmed by TCA-precipitation followed by SDS-PAGE analysis. Recombinant NFS1-ISD11-ISCU complex co-purified with *E. coli* ACP, and will hereafter be referred to as the NFS1-ISD11-ACP-ISCU (SDAU) complex.

### 2.4 Differential scanning fluorimetry

Miniaturized DSF (nanoDSF) was performed in 10 μL capillaries using the Prometheus device (NanoTemper Technologies) that uses excitation at 280 nm to measure emission from tryptophan and tyrosine residues at two wavelengths: 330 (non-polar environment emission) and 350 nm (polar environment emission). Each capillary consists of ISCU protein at 0.8 mg/mL in buffer containing 50 mM HEPES pH 7.5, 200 mM NaCl and 10 mM DTT, supplemented with either 300 μM EDTA or 100 μM ZnCl_2_. Thermal unfolding was carried out using a linear thermal ramp (1.0 °C/min; 20 °C–95 °C) and unfolding midpoint (melting temperature, T_m_) was determined from changes in the emission wavelengths of tryptophan and tyrosine fluorescence at 330 and 350 nm.

### 2.5 Circular dichroism

Circular dichroism (CD) spectra were recorded on a J-815 spectropolarimeter (JASCO) at 20 °C with a scan speed of 100 nm/min using 0.1 cm pathlength quartz cells from Starna Scientific UK. The concentration of ISCU protein was at 0.1 mg/mL in buffer which consisted of 50 mM HEPES pH 7.5, 200 mM NaCl, 5 mM DTT and either 100 μM EDTA or 30 μM ZnCl_2_ and then buffer exchanged into 10 mM Tris pH 7.5 (the pH was adjusted with phosphoric acid) and 50 mM NaF before acquiring data. Data points were collected with a resolution of 0.2 nm, an integration time of 1 s, and a slit width of 1 nm. Each spectrum shown is the result of nine averaged consecutive scans, from which buffer scans were subtracted.

### 2.6 BioLayer interferometry (BLI)

BLI experiments were performed on a 16-channel ForteBio Octet RED384 instrument at 25 °C, in buffer containing 50 mM HEPES pH 7.5, 200 mM NaCl, 5% Glycerol, 2 mM TCEP, 5 mM DTT, 0.5 mg/mL BSA, which is further supplemented with either 100 μM ZnCl_2_ or 300 μM EDTA. 50 μL of 1 mg/mL of biotinylated ISCU was diluted to 650 μL and loaded to the streptavin coated sensors. The concentration for SDA used ranged from 10 μM to 1.56 nM. Measurements were performed using a 300 s association step followed by a 300 s dissociation step on a black 384-well plate with tilted bottom (ForteBio). The baseline was stabilized for 30 s prior to association and signal from the reference sensors was subtracted. A plot of response vs. [NFS1] was used for *K*_*d*_ determination using one site-specific binding fit in GraphPad Prism (GraphPad Software).

## 3 Results

### 3.1 Recombinant ISCU is purified as a mixture of zinc-depleted and zinc-bound forms

In frataxin-deleted yeast, a substitution on the scaffold protein Isu at position Met141 to either Cys, Ile, Leu, or Val, corrected the loss-of-frataxin phenotypes and rescued cell viability [33,34]. In the human ISCU protein (167-aa precursor protein; 133-aa mature protein without the mitochondrial signal peptide) which bears 72% sequence identity to yeast (Supplemental Fig. 1), the equivalent residue is Met140 (previously referred to as Met106). This study sets out to determine if recombinant human ISCU, harboring the Met140 variants, would modulate desulfurase activity similar to the observations made in yeast [[32], [33], [34]]. As the first step, we generated recombinant human ISCU wild-type and variants (ISCU_M140I_, ISCU_M140L_ and ISCU_M140V_) in order to compare their biochemical and biophysical properties.

**Fig. 1:**
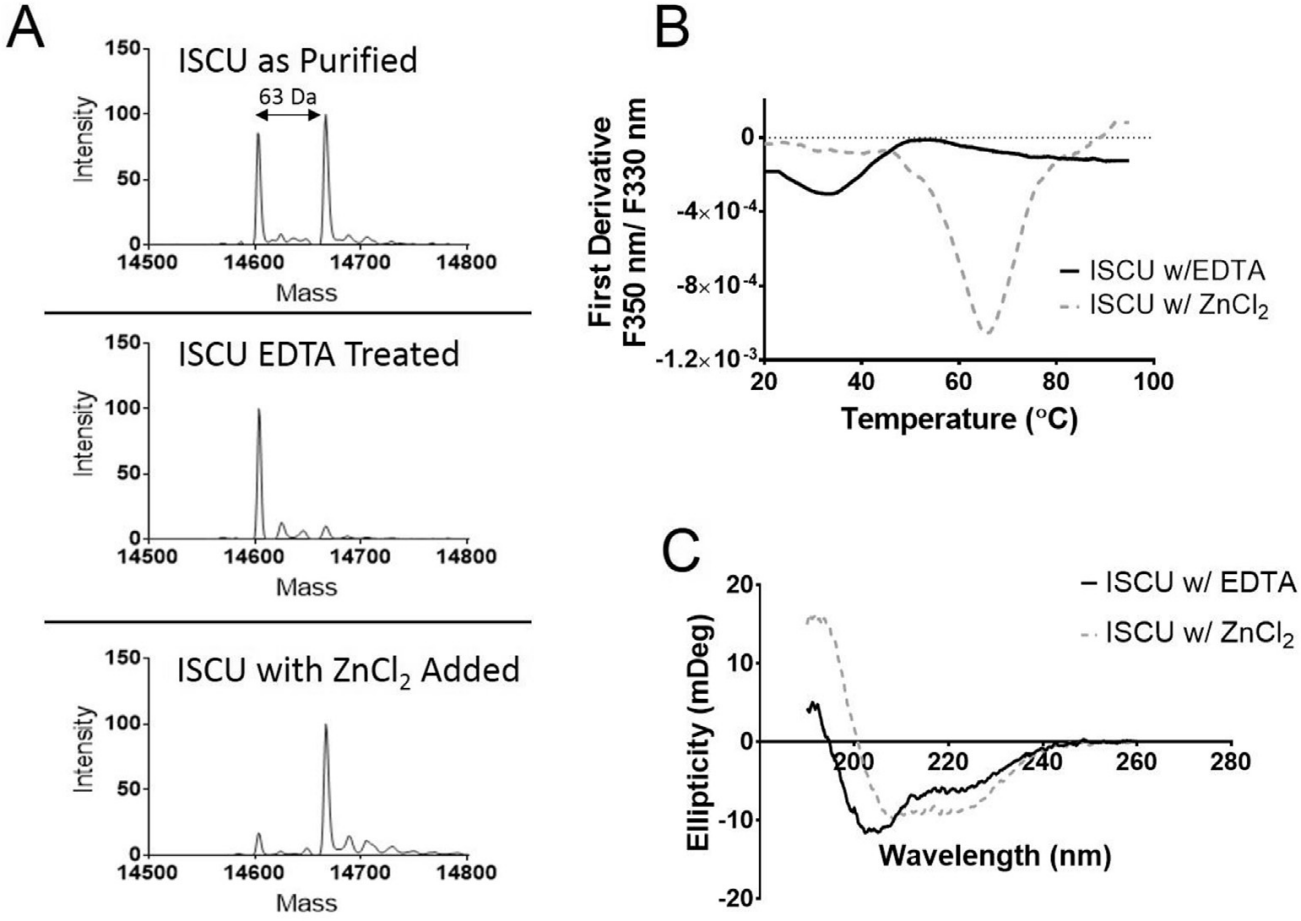
**Characterization of wild-type ISCU in zinc-depleted and zinc-bound forms**. (A) Mass Spectrometry analysis of purified ISCU shows the existence of two states with a mass difference of 64 Da consistent with one zinc(II) ion being bound. EDTA can successfully remove the zinc, and on the contrast the addition of ZnCl_2_ can push it to the zinc-bound state. (B) To determine thermostability of the two states, nanoDSF was used to determine the melting temperatures showing that zinc-bound was ∼30 °C higher than that for the zinc-depleted form (T_m_ of 65.7 ± 0.2 °C, 33.2 ± 0.6 °C, respectively) and thus much more stable. (C) Circular Dichroism was used to determine secondary structure of the two states and found that the zinc-bound state had a higher helical percentage and a lower disordered percentage than that of the zinc-depleted state.

During purification we observed by native mass spectrometry that recombinant ISCU protein exists in two forms, one corresponding to the expected mass of the apo-protein (for ISCU_WT_ 14604 Da), and the other with an additional mass of 64 Da (14668 Da), which suggests a subset of ISCU is zinc-bound form with one zinc(II) ion per ISCU monomer (Fig. 1A top). Similar observations were also made with the ISCU_M140I_, ISCU_M140L_ and ISCU_M140V_ variants. ISCU was treated with either EDTA or supplemented with ZnCl_2_ and the absence or presence of the metal was analysed by native mass spectrometry (Fig. 1A for wild-type; Supplemental Fig. 2A and B for variants). NanoDSF demonstrated that zinc-bound ISCU (WT/variants) is significantly more thermostable with a melting temperature ∼30 °C higher than that for the zinc-depleted form (for ISCU_WT_, T_m_ of 65.7 ± 0.2 °C *vs* 33.2 ± 0.6 °C) (Fig. 1B, Table 1). The metal-conferred thermostability was also reflected by far-UV circular dichroism spectra consistent with zinc-bound ISCU displaying a higher helical content and less disordered content than the zinc-depleted ISCU (Fig. 1C for wild-type; Fig. 3A for variants). The observed changes in secondary structure possibly occur around the Fe-S cluster binding site. Such alterations were previously reported bacterial structures of IscU with and without zinc [7,24].

**Fig. 2:**
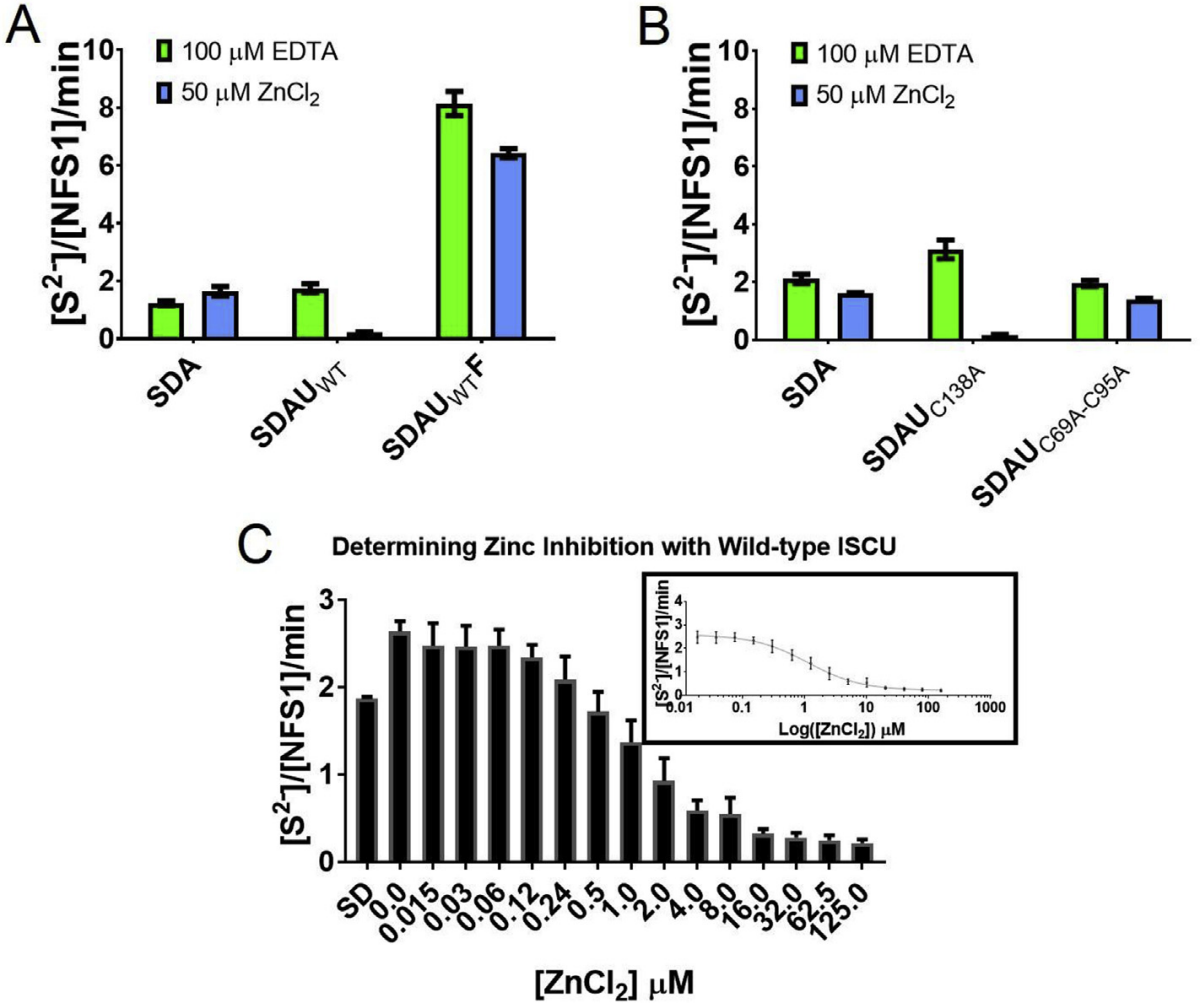
**Activity assay analysis of wild-type ISCU in zinc-depleted and zinc-bound forms**. (A) The methylene blue assay was used to determine the effect of desulfurase activity on the two states of ISCU using buffer supplemented with either 100 μM EDTA or 50 μM ZnCl_2_. When noted, concentration of NFS1-ISD11-ACP (SDA) was at 0.5 μM, ISCU (U) at 2.5 μM, and Frataxin (F) at 40 μM. (B) The three conserved cysteine residues on ISCU were mutated to alanine (single variant C138A, double variant C69A-C95A) to determine if there was still a zinc-based inhibition of desulfurase activity. (C) In order to determine IC_50_ values for zinc to the NFS1-ISD11-ACP-ISCU (SDAU) complex, we treated ISCU with EDTA to remove any zinc and then buffer exchanged to remove EDTA into buffer without EDTA or ZnCl_2_. The methylene blue assay was then used in the presence of buffer containing a serial dilution of zinc concentrations and plotting [inhibitor] *vs.* response (inset shown on a log scale) to determine an IC_50_ of 0.90 ± 0.06 μM for ZnCl_2_ to SDAU. Error bars denote standard deviations determined from experimental replicates of n = 3.

**Fig. 3:**
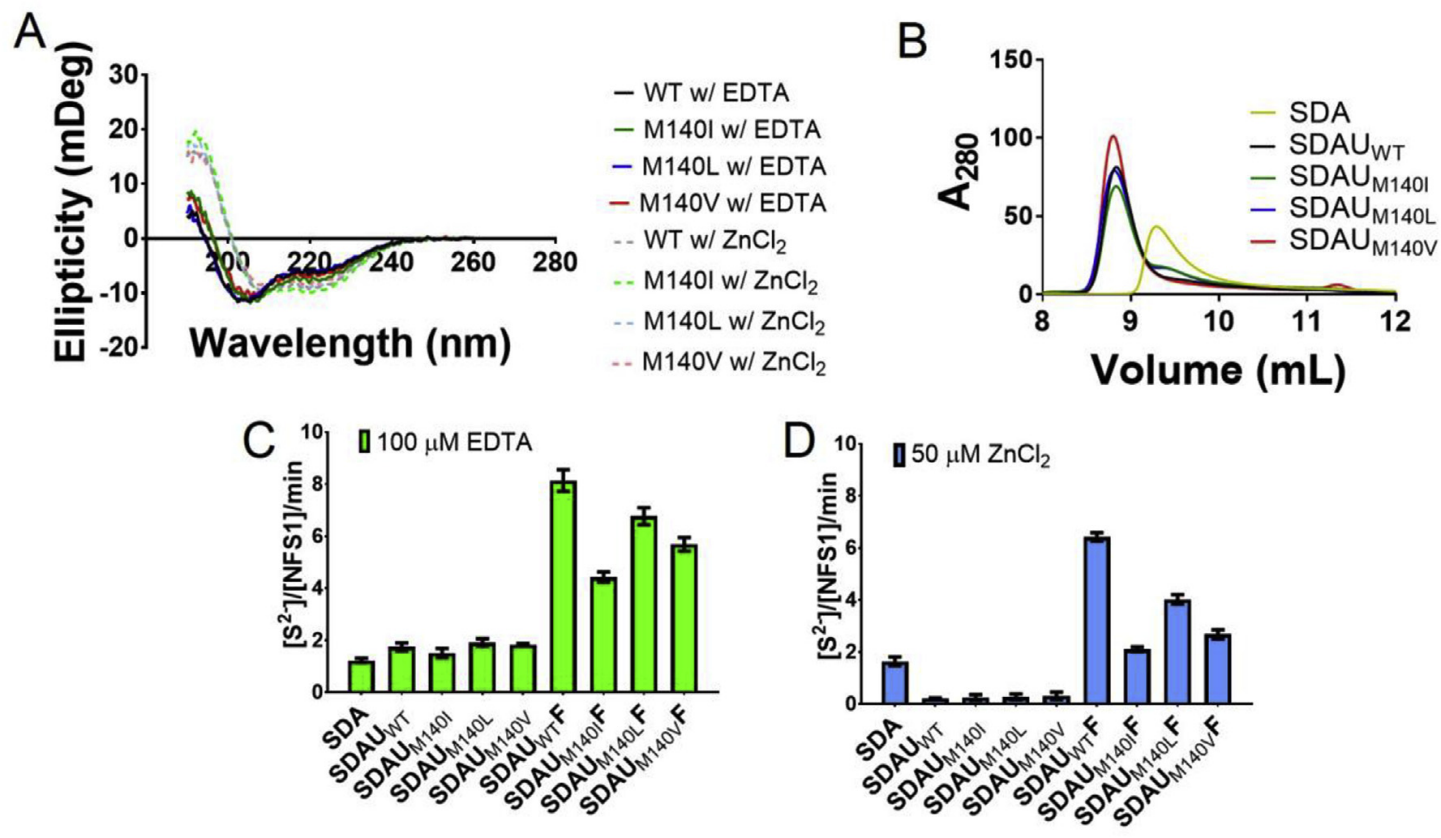
**Comparison of biophysical characteristics of wt ISCU and M140 mutants, and their corresponding effect on complex activity**. (A) Circular Dichroism was used to determine secondary structure of the two states of ISCU and compare the WT to the M140I, M140L, and M140V variants and found no difference between them and showed similarly that the zinc-bound state had a higher helical percentage and a lower disordered percentage than that of the zinc-depleted state. (B) Analytical gel filtration was used to determine if complex formation was intact for NFS-ISD11-ACP (SDA), NFS1-ISD11-ACP-ISCU_WT_ (SDAU_WT_), NFS1-ISD11-ACP--ISCU_M140I_ (SDAU_M140I_), NFS1-ISD11-ACP-ISCU_M140L_ (SDAU_M140L_), and NFS1-ISD11-ACP-ISCU_M140V_ (SDAU_M140V_). (C, D) The methylene blue assay was used to determine the effect of desulfurase activity on the two states of ISCU WT and variants using buffer supplemented with either 100 μM EDTA (C) or 50 μM ZnCl_2_ (D). When noted, concentration of NFS1-ISD11-ACP (SDA) was at 0.5 μM, ISCU (U) at 2.5 μM, and Frataxin (F) at 40 μM. For (C) and (D) error bars denote standard deviations determined from experimental replicates of n = 3.

**Table 1:** A Comparison of the Biophysical Properties of WT-ISCU and Proposed Suppressor Variants. Standard deviations were determined from experimental replicates of n = 3.

Variant	Additive	[S^2−^]/[NFS1]/min Without FXN	[S^2−^]/[NFS1]/min With FXN	ZnCl_2_ IC_50_ (μM)	T_m_ (°C)	*K*_*d*_ (nM) (BLI of SDA-U)
WT	EDTA	1.75 ± 0.16	8.14 ± 0.42	NA	33.2 ± 0.6	87.8 ± 7.0
	ZnCl_2_	0.21 ± 0.02	6.42 ± 0.16	0.90 ± 0.06	65.7 ± 0.2	160 ± 26
M140I	EDTA	1.51 ± 0.18	4.43 ± 0.20	NA	34.2 ± 0.7	97.3 ± 8.7
	ZnCl_2_	0.25 ± 0.10	2.11 ± 0.09	1.71 ± 0.14	64.7 ± 0.4	194 ± 31
M140L	EDTA	1.92 ± 0.14	6.77 ± 0.33	NA	30.9 ± 0.2	110 ± 11
	ZnCl_2_	0.27 ± 0.11	4.02 ± 0.18	0.52 ± 0.09	63.5 ± 0.2	181 ± 28
M140V	EDTA	1.83 ± 0.05	5.69 ± 0.26	NA	34.3 ± 0.5	85.5 ± 6.8
	ZnCl_2_	0.32 ± 0.15	2.67 ± 0.17	0.90 ± 0.07	64.6 ± 0.3	218 ± 30

### 3.2 ISCU modulates NFS1 desulfurase activity *in vitro* in a zinc-dependent manner

In the first steps of Fe-S cluster assembly, ISCU functions as a scaffold protein for the NFS1-ISD11-ACP desulfurase complex (SDA), which generates and transfers the inorganic sulfur to ISCU, in a reaction activated by FXN protein [3,39]. We tested if the presence of zinc(II) ion in purified samples influences NFS1 desulfurase activity using a methylene blue activity assay that quantifies the amount of sulfide produced [3,38,40,41], in reaction buffer supplemented with either EDTA (100 μM) or ZnCl_2_ (50 μM) (Fig. 2A). SDA displayed a basal level of desulfurase activity in both buffers (1.23 ± 0.08 mol/NFS1/min in EDTA-containing buffer; 1.64 ± 0.17 mol/NFS1/min in ZnCl_2_-containing buffer). The addition of ISCU to NFS1-ISD11-ACP (SDAU) increased the desulfurase activity by ∼1.5 fold when assayed in EDTA-containing buffer. To our surprise, when SDAU was assayed in ZnCl_2_-containing buffer, desulfurase activity was almost completely abolished. The activity of SDA alone, without ISCU, was not affected by increasing ZnCl_2_ concentration (Fig. 2A, Supplemental Fig. 3), indicating that the zinc effect is mediated directly through the ISCU protein, and not due to indirect solution properties (e.g. metal-induced aggregation of the SDA sample) [42]. As expected, further addition of the FXN activator (at 80 equivalents) to SDAU (forming SDAUF) increased desulfurase activity in both buffer conditions, reaching a maximal activity of 8.14 ± 0.42 mol/NFS1/min in EDTA-containing buffer (4.5-fold increase from SDAU) and 6.42 ± 0.16 mol/NFS1/min in ZnCl_2_-containing buffer (30-fold increase from SDAU) (Fig. 2A, Table 1).

The Cys138 residue on ISCU has been shown to be the persulfide acceptor from NFS1 [36]. We tested if this residue was involved in the zinc-mediated inhibition and thus bound to the zinc (II) ion. We constructed the C138A variant of ISCU and discovered that cysteine desulfurase activity of SDAU_C138A_ can still be inhibited in the presence of zinc (Fig. 2B). We further constructed the C69A-C95A double variant of ISCU (where the only conserved cysteine residue remaining is Cys138), which showed very little zinc-based inhibition on the desulfurase activity of SDAU_C69A-C95A_. Therefore Cys138 is not involved in zinc(II) ion coordination. Altogether, our results strongly indicate that the inhibition of SDA desulfurase activity by ISCU *in vitro*, an observation previously reported by various groups [3,20,39,43], is a function of the divalent zinc(II) ion.

To quantify zinc-mediated inhibition, ISCU was pre-incubated with EDTA to first remove any bound zinc, and then buffer was exchanged into EDTA-free buffer with increasing ZnCl_2_ concentrations, prior to assaying for desulfurase activity (Fig. 2C). Increasing amounts of zinc(II) ion demonstrated a dose-dependent inhibition of desulfurase activity with an IC_50_ of 0.90 μM ± 0.06 (Fig. 2C). To determine if the inhibition of SDA activity was due to disrupted protein-protein interactions between ISCU and the SDA complex, we characterized the binding between SDA and biotinylated ISCU using BioLayer interferometry (BLI) (Table 1, Supplemental Fig. 4). We did not observe any significant difference between the zinc-bound and zinc-depleted forms of ISCU in their binding to SDA, with dissociation constants in the nanomolar range: 87.8 ± 7.0 nM in buffer containing EDTA and 160 ± 26 nM in buffer containing ZnCl_2_. Hence complex formation was not affected by either form of ISCU.

### 3.3 Human ISCU variants M140I, M140L and M140V behave similar to wild-type *in vitro*

Like wild-type protein, the ISCU_M140I_, ISCU_M140L_ and ISCU_M140V_ variants were purified as a mixed population of interconvertible zinc-depleted and zinc-bound forms. All variants behaved like ISCU_WT_ in terms of secondary structure properties as analysed by CD (Fig. 3A, Table 1), zinc-mediated thermostability from nanoDSF (Table 1), and the ability to form a stable complex with SDA in size exclusion chromatography (Fig. 3B, Supplemental Fig. 5) and BLI (Table 1, Supplemental Fig. 4). Altogether, recombinant ISCU wildtype and variant proteins have indistinguishable structural and biophysical properties.

We next employed the methylene blue activity assay to determine the effect of ISCU variants on the SDA complex activity. All ISCU variants behaved similar to wild-type in their respective zinc-depleted forms, and increased activity slightly by ∼1.5 fold compared to SDA-alone (Fig. 3C, Table 1). Importantly, in zinc-bound form, SDA activity was once again abolished (Fig. 3D, Table 1). Therefore, the ISCU variants inhibited SDA activity in a zinc-dependent manner, with IC_50_ values for ZnCl_2_ within the same order of magnitude as observed for SDA in the presence of wild-type ISCU (Table 1, Supplemental Fig. 6). The SDAU complexes containing ISCU variants retained the ability to be activated by frataxin under both buffer conditions tested, with the degree of activation (evaluated by the maximal desulfurase activity reached in each case) in the following decreasing order: ISCU_WT_ (i.e. SDAU_WT_F complex) > ISCU_M140L_ (i.e. SDAU_M140L_F complex) > ISCU_M140V_ (i.e. SDAU_M140V_F complex) > ISCU_M140I_ (i.e. SDU_M140I_F complex) (Fig. 3C and D, Table 1).

## 4 Discussion

In this study we set out to characterize recombinant human ISCU wild-type and variant proteins for their functional interaction with SDA and effect on NFS1 activity. Our biophysical characterization of human ISCU is consistent with the existence of two conformational states, one being structured and one disordered around the Fe-S cluster binding site [44]. In fact *E. coli* IscU was previously shown to exist in these two interconvertible conformations that can be influenced by interaction with zinc [22,25,26]. Several structures of bacterial IscU (75% sequence identity to human ISCU) also reveal a more structured conformation such as when having an Fe-S cluster bound (PDB: 2Z7E) [27], in complex with IscS (PDB: 3LVM) [28,29], carrying a substitution on Asp39 (E.coli numbering, human equivalent Asp71; PDB: 2KQK) [24], or, most commonly, when bound to zinc(II) ion in place of an Fe-S cluster via the conserved cysteine residues (e.g. PDB: 1WFZ, 1RP9P, 1XJS, 1SU0) [30,31]. The more flexible/disordered conformation is observed in an NMR structure of wild type *E. coli* apo-IscU (PDB: 2L4X) undergoing a helix-to-coil rearrangement in the helix containing the sulfur acceptor residue Cys106 (E.coli numbering, human equivalent Cys138) [24].

Previous publications have shown that ISCU, in the absence of frataxin, inhibits the NFS1 desulfurase activity *in vitro* [36,39,45]. Our study attributes the inhibitory property of ISCU to the presence of zinc(II) ions bound to ISCU protein from recombinant expression, likely associated with the active site cysteine residues (as observed in several crystal structures of zinc-bound bacterial IscU). Our data reveal no significant difference between the two ISCU forms in their binding affinity to the SDA complex. One possible explanation for the zinc-mediated inhibition is that the bound zinc(II) ion could inhibit NFS1 activity by locking the NFS1 mobile loop active site cysteine. Consistent with this, the recently published crystal structures of SDAU, in the presence and absence of zinc [7], reveal the zinc(II) ligation to include three ISCU residues (Asp 71, Cys95, and His137, human precursor numbering) and the mobile loop Cys381 from NFS1 [7]. This would imply that the mobile loop cysteine on NFS1 is sequestered by zinc away from the PLP cofactor, blocking sulfur flux from NFS1, and resulting in inhibition of NFS1 activity. We showed that FXN can activate the SDAU complex in the presence or absence of zinc, suggesting that FXN can cause a conformation change on ISCU and/or NFS1 to release the functional cysteine residues and initiate sulfide production. This is supported by recent evidence that zinc can modulate the Fe-S cluster assembly process in the B. subtilis sulfur mobilization system, whereby it stabilizes the scaffold protein SufU upon binding to cysteine desulfurase SufS [46].

The zinc(II) ion observed in our ISCU samples is likely derived from recombinant expression in *E. coli*, a phenomenon that may have occurred during sample preparation for previous *in vitro* studies of recombinant E coli IscU and human ISCU [36,39,45]. Some variations in the finite ratio of zinc-bound to zinc-depleted populations exist among our different preparations of recombinant ISCU. Although the majority of as-purified ISCU protein is in the zinc-bound form, upon EDTA treatment, can be converted to the zinc-depleted form resulting in modest activation of NFS1 activity. We therefore reason that previous characterization of ISCU/IscU biochemical and biophysical properties [36,39,45] should be interpreted with caution, and in the context that as-purified ISCU/IscU would potentially be present in both zinc-bound and zinc-depleted forms, influencing the equilibrium between ISCU conformations. Whether this also happens in vivo, and has biological relevance for regulation of Fe-S cluster biosynthesis, is currently unknown. It is of note that in human cells, the total concentration of zinc is 200–300 μM [47], with the major pool of zinc residing in the mitochondria and free zinc uptake in human mitochondria reported to be in the range of 80 pm–20 μM [48]. Therefore, a physiologically-relevant role for zinc could exist in vivo, whereby the bound zinc(II) ion could play a role in modulating Fe-S metabolism and oxidative stress in mitochondria, although this clearly warrants investigation in future studies.

Another objective of this study was to determine, using our recombinant expression system, the effect of substituting Met140 with Ile, Leu, and Val in ISCU on its biochemical and biophysical properties. The equivalent substitution in yeast Isu yielded recovery of growth and cell viability defects in cells deficient in frataxin Yfh1. Additionally, using purified yeast proteins, the IscU suppressor has been shown to activate Nfs1 and bypass the requirement for Yfh1 *in vitro* [35]. Our data suggest that the human ISCU suppressor variants, when recombinantly expressed in *E. coli*, were essentially indistinguishable from wild-type with regards to zinc binding, secondary structure, thermostability, and complex formation. Importantly, under both zinc-replete and zinc-depleted buffer conditions tested, there is negligible desulfurase activity for the frataxin-free SDAU complex with either ISCU wild-type or Met140 variants. The SDAU complexes containing ISCU variants retained the ability to be activated by frataxin under both buffer conditions tested but observed a trend in desulfurase activity with the elongated and less branched side chains being most active (ISCU_WT_ > ISCU_M140L_ > ISCU_M140V_ > ISCU_M140I_ (Fig. 3C and D, Table 1)). We reason that a substitution at the Met140 position might decrease the ability for DTT to release the sulfide from ISCU variants compared to WT, or interfere with the capacity of human FXN to fully activate the complex.

Our inability to observe a FXN bypass effect with the human ISCU suppressor mutants may be due to differences between the yeast and human proteins, or due to a number of factors including assays and reagents, and potentially variable levels of zinc(II) ion that were not controlled by other studies [[32], [33], [34],^38^]. For example, human FXN cannot activate NFS1 without ISCU present [18], while yeast Yfh1 can in the absence of Isu1 (and sometimes without Isd11) [35]. Additionally, different activity assays were used to quantify NFS1 desulfurase activity in this study compared to the previous study reporting FXN bypass [35]. Here, a colorimetric assay was used to measure sulfide production and release in the presence of a reductant, while in the previous study sulfide accumulation on NFS1 was probed with [^35^S]-Cys by scintillation counting and autoradiography. Importantly, this study has demonstrated that additional factors present in the purified samples, for example zinc(II) ion, can directly influence NFS1 activity. Uncontrolled variability in zinc content and its impact on reported complex activities between studies, and even between batches of protein from within one study, may be an important factor that contributed to the discrepancy between studies. In summary, the complexity of the multi-component Fe-S assembly system and the level of zinc(II) ion co-purified with ISCU need to be taken into account in future experimental design when exploring human SDAU/SDAUF complex activity.

## Footnotes

1 In the literature, residues of human ISCU or yeast Isu residues have often been numbered by referring to the mature protein without the N-terminal mitochondrial targeting sequence. For clarity among the general scientific community, we hereafter refer to residues of human ISCU or yeast Isu based on numbering of the full-length translated protein (UNIPROT entry Q9H1K1 or Q03020). Both numbering schemes were included in the first mention of the residue in the main text.
