# Supplementary material for: Zinc(II) binding on human wild-type ISCU and Met140 variants modulates NFS1 desulfurase activity
Source: Biochimie. 2018 Sep;152:211–8. doi: 10.1016/j.biochi.2018.07.012 (PMC6098246; doi:10.1016/j.biochi.2018.07.012)

**Zinc(II) binding on human wild-type ISCU and Met140 variants modulates NFS1 desulfurase activity**

Nicholas G. Fox,^†^ Alain Martelli,^‡^ Joseph F. Nabhan,^‡^ Jay Janz,^‡^ Oktawia Borkowska,^†^ Christine Bulawa,^‡,*^ and Wyatt W. Yue^†,*^

^†^Structural Genomics Consortium, Nuffield Department of Clinical Medicine, University of Oxford, UK OX3 7DQ

^‡^Pfizer Rare Disease Research Unit, Worldwide Research and Development, Pfizer Inc., 610 Main Street, Cambridge, Massachusetts 02140

^*^Corresponding author: Wyatt Yue, Phone: +44 (1865) 617757, Email: [wyatt.yue@sgc.ox.ac.uk](mailto:wyatt.yue@sgc.ox.ac.uk)

^*^Corresponding author: Christine Bulawa, Phone: 781-697-7482, Email: [christine.bulawa@pfizer.com](mailto:christine.bulawa@pfizer.com)

**Supplementary Material TOC**

**Supplemental Figure 1:** Sequence alignment of human ISCU to *E. coli* and yeast orthologs.

**Supplemental Figure 2:** Mass Spectrometry analysis of the ISCU variants in zinc-depleted and zinc-bound forms.

**Supplemental Figure 3:** Activity assay of NFS1-ISD1-ACP with zinc.

**Supplemental Figure 4:** Determine binding constants of WT-ISCU and variants to NFS1-ISD11-ACP using BioLayer Interferometry (BLI).

**Supplemental Figure 5:** SDS-PAGE analysis of complex formation from analytical gel filtration analysis.

**Supplemental Figure 6:** Determining IC_50_ values of zinc inhibition for ISCU WT and variants.


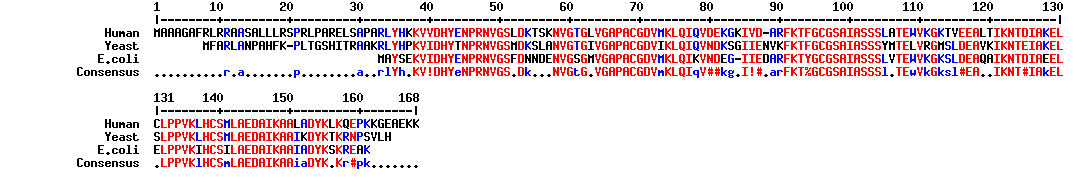


**Supplemental Figure 1: Sequence alignment of human ISCU to *E. coli* and yeast orthologs.** Sequence alignment shows human to *E. coli* is 75% identity and human to yeast is 72% identity.


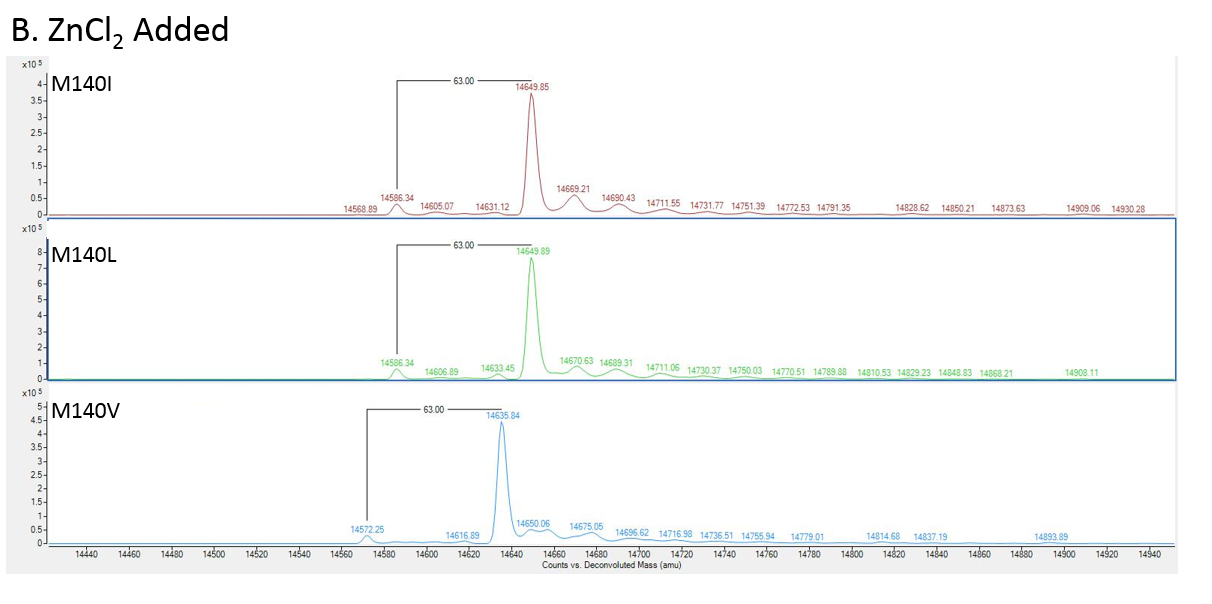


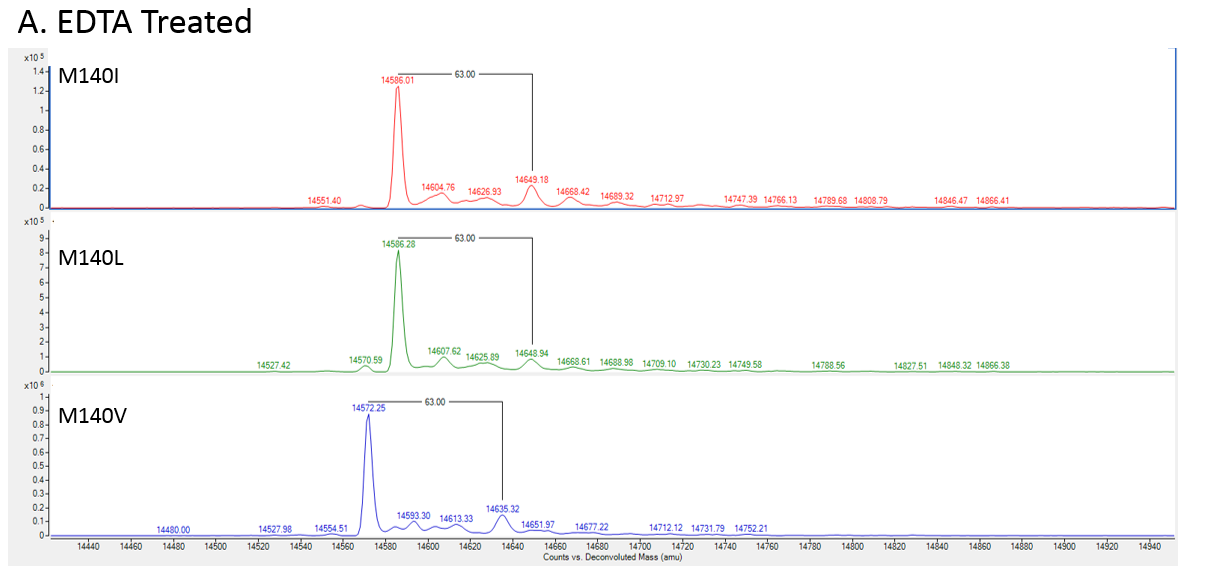


**Supplemental Figure 2: Mass Spectrometry analysis of the** **ISCU variants in zinc-depleted and zinc-bound forms.** Mass Spectrometry analysis of EDTA treated (A) and ZnCl_2_ treated (B) ISCU variants. The mass difference of 64 Da is consistent with a zinc ion being absent or present, respectively.

**Supplemental Figure 3: Activity assay of NFS1-ISD11 with zinc.** The methylene blue assay was used to determine the effect of desulfurase activity upon the addition of zinc. The concentration of NFS1-ISD11-ACP (SDA) was at 0.5 μM and three zinc concentrations 0, 4, and 125 μM were used as a control to show zinc was not affecting SDA or the activity assay.

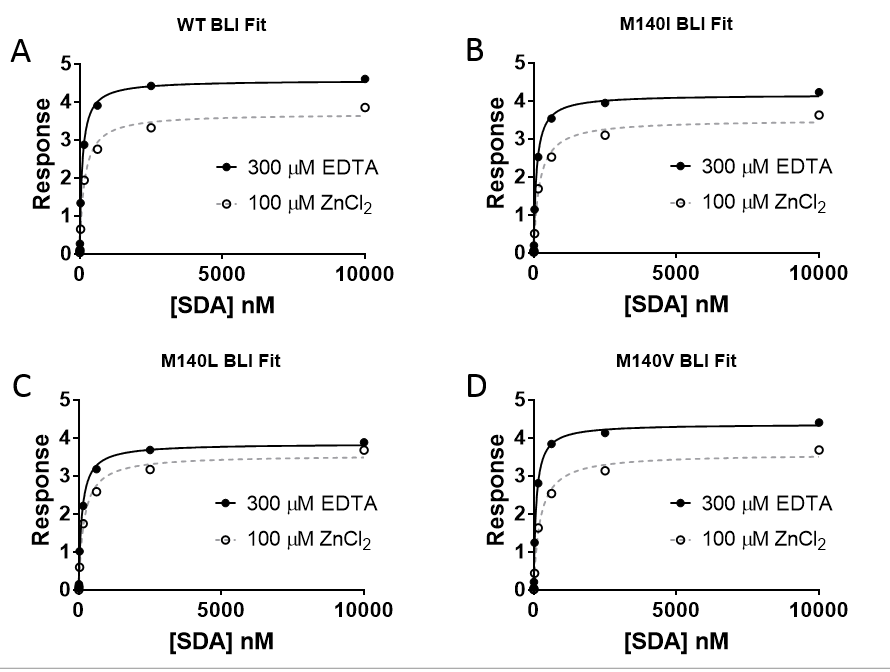


**Supplemental Figure 4: Determine binding constants of WT-ISCU and variants to NFS1-ISD11-ACP using BioLayer Interferometry (BLI).** A plot of response vs. [SDA] was used for *K_d_* determination using one site-specific binding fit in GraphPad Prism to determine the binding of biotinylated ISCU to NFS1-ISD11-ACP for WT-ISCU (A), M140I-ISCU (B), M140L-ISCU (C), and M140V-ISCU (D).


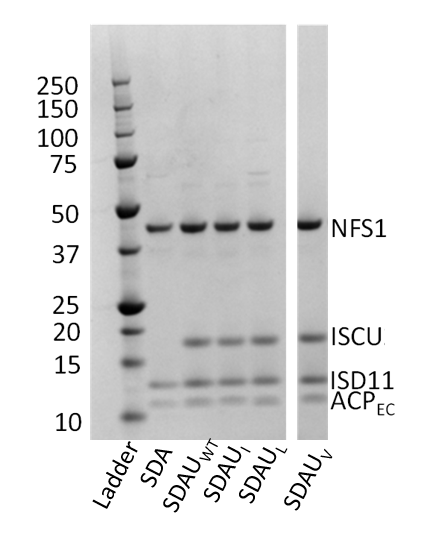


**Supplemental Figure 5: SDS-PAGE analysis of complex formation from analytical gel filtration analysis.** Samples from Figure 3B were TCA precipitated and run on an SDS-PAGE gel for analysis. Complex formation was successful for NFS-ISD11-ACP (SDA), NFS1-ISD11-ACP-ISCU_WT_ (SDAU_WT_), NFS1-ISD11-ACP-ISCU_M140I_ (SDAU_I_), NFS1-ISD11-ACP-ISCU_M140L_ (SDAU_L_), and NFS1-ISD11-ACP-ISCU_M140V_ (SDAU_V_) and all had an *E. coli* contaminating protein, the acyl carrier protein (ACP_EC_).

**Supplemental Figure 6: Determining IC_50_ values of zinc inhibition for ISCU WT and variants.** The methylene blue assay was used to determine IC_50_ values for zinc to the NFS1-ISD11-ACP-ISCU (SDAU) complexes of WT (A), M140I (B), M140L (C), and M140V (D) by using buffer containing a serial dilution of zinc concentrations and plotting [inhibitor] *vs.* response (inset shown on a log scale).


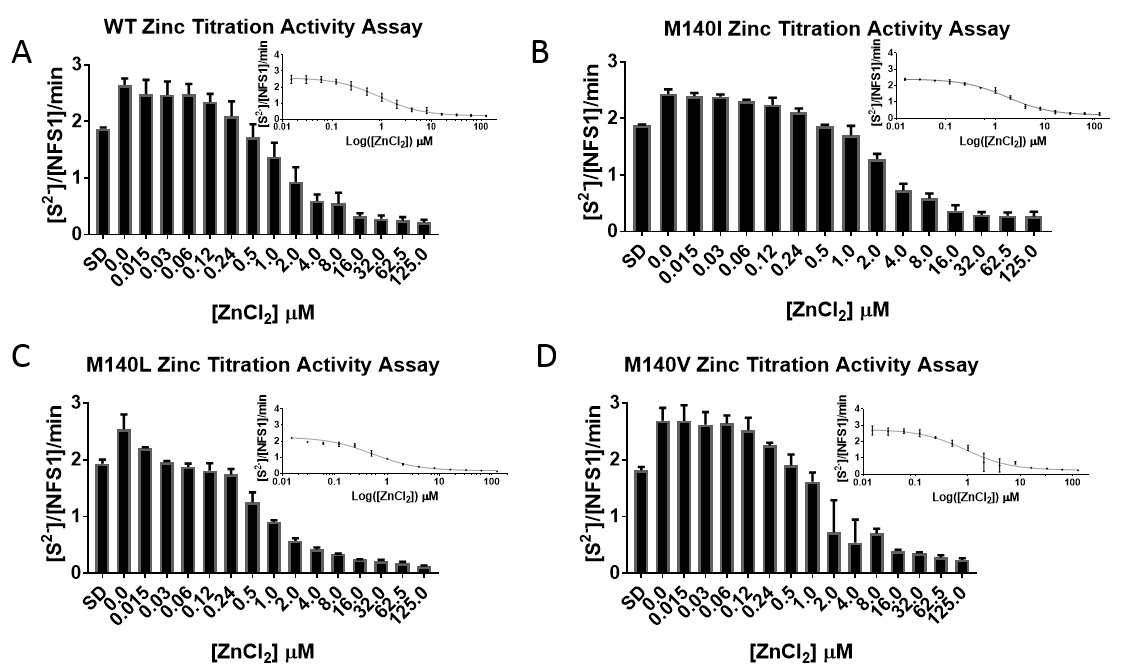

Supplement: Supplementary material [file mmc1.docx]
